# Supplementary material for: The micro-niche explains allotopy and syntopy in South American Liolaemus (Iguania: Liolaemidae) lizards
Source: PeerJ. 2025 Feb 17;13:e18979. doi: 10.7717/peerj.18979 (PMC11841597; doi:10.7717/peerj.18979)
Supplement: Supplemental Information 1 — TEMP (Temperature); HUM (Relative Humidity); WS (Wind Speed); SR (Sun radiation). Values correspond to mean ± standard deviation. Letters at the right identify significate differences. Similar letters mean no difference. [file peerj-13-18979-s001.pdf]

Supplementary Table 1. Pos hoc probabilities of the comparisons of the sites between months made for the variables studied. TEMP (Temperature); HUM (Relative Humidity); WS (Wind Speed); SR (Sun radiation). Values correspond to mean ± standard deviation. Letters at the right identify significate differences. Similar letters mean no difference.

JAN				
	TEMP	HUM	WS	SR
SITE 1	16.4 ± 0.19 A	36.9 ± 1.65 A	2.2 ± 0.04 A	559.26 ± 161.03
SITE 2	15.3 ± 0.20 B	44.7 ± 2.01 B	2.0 ± 0.06 B	559.29 ± 158.80
SITE 3	16.9 ± 0.21 A	44.1 ± 1.97 B	1.9 ± 0.05 C	555.82 ± 158.48
SITE 4	15.1 ± 0.21 B	45.4 ± 2.03 B	2.0 ± 0.06 B	560.11 ± 159.07
P	< 0.0001	< 0.0001	<0.001	>0.05
FEB				
	TEMP	HUM	WS	SR
SITE 1	15.8 ± 0.16 A	38.5 ± 0.19 A	2.1 ± 0.01 A	539.23 ± 163.73
SITE 2	14.6 ± 0.16 B	46.7 ± 0.25 B	1.9 ± 0.01 B	533.77 ± 162.92
SITE 3	16.2 ± 0.18 A	45.9 ± 0.23 B	1.8 ± 0.01 C	535.60 ± 163.09
SITE 4	14.4 ± 0.17 B	47.4 ± 0.25 B	1.9 ± 0.01 B	536.68 ± 162.86
P	< 0.0001	< 0.0001	< 0.0001	>0.05
MAR				
	TEMP	HUM	WS	SR
SITE 1	14.9 ± 0.49 A	36.5 ± 1.24 A	2.0 ± 0.02 A	541.38 ± 182.91
SITE 2	13.8 ± 0.47 B	44.3 ± 1.45 B	1.9 ± 0.02 B	540.99 ± 182.72
SITE 3	15.2 ± 0.71 A	44.2 ± 2.65 B	1.8 ± 0.09 C	540.39 ± 182.79
SITE 4	13.5 ± 0.47 B	45.0 ± 1.45 B	1.9 ± 0.02 B	538.74 ± 185.48
P	< 0.0001	< 0.0001	< 0.0001	>0.05
APR				
	TEMP	HUM	WS	SR
SITE 1	13.1 ± 0.55 A	30.1 ± 2.18 A	1.9 ± 0.03 A	493.83 ± 190.35
SITE 2	11.9 ± 0.58 B	37.0 ± 2.50 B	1.8 ± 0.03 B	492.71 ± 189.85
SITE 3	13.4 ± 0.59 A	36.8 ± 2.46 B	1.7 ± 0.03 C	493.18 ± 190.18
SITE 4	11.7 ± 0.58 B	37.6 ± 2.53 B	1.8 ± 0.03 B	492.43 ± 189.79
P	< 0.0001	< 0.0001	< 0.0001	>0.05
MAY				
	TEMP	HUM	WS	SR
SITE 1	10.7 ± 0.89 A	24.2 ± 1.11 A	1.9 ± 0.03 A	449.06 ± 192.31
SITE 2	9.5 ± 0.85 B	30.2 ± 1.23 B	1.8 ± 0.03 B	447.61 ± 191.56
SITE 3	10.9 ± 0.88 A	30.1 ± 1.23 B	1.7 ± 0.03 C	448.75 ± 191.88
SITE 4	9.13 ± 1.14 A	31.2 ± 1.34 B	1.8 ± 0.03 B	447.19 ± 191.47
P	< 0.0001	< 0.0001	< 0.0001	>0.05
JUN				
	TEMP	HUM	WS	SR
SITE 1	8.1 ± 0.45 A	21.1 ± 0.42 A	2.04 ± 0.03 A	410.16 ± 186.23
SITIO 2	6.9 ± 0.45 B	27.9 ± 0.51 B	1.9 ± 0.03 B	409.80 ± 185.93
SITE 3	8.4 ± 0.44 A	27.8 ± 0.52 B	1.8 ± 0.03 C	410.77 ± 185.70
SITE 4	6.6 ± 0.45 B	28.4 ± 0.51 B	1.9 ± 0.03 B	409.37 ± 185.88
P	< 0.0001	< 0.0001	< 0.0001	>0.05
JUL				
	TEMP	HUM	WS	SR
SITE 1	8.3 ± 0.43 A	20.5 ± 0.46 A	2.1 ± 0.01 A	441.28 ± 195.66
SITE 2	7.1 ± 0.41 B	25.7 ± 0.63 B	1.9 ± 0.01 B	441.64 ± 195.68
SITE 3	8.5 ± 0.44 A	25.5 ± 0.65 B	1.8 ± 0.01 C	442.79 ± 195.94
SITE 4	6.8 ± 0.44 B	26.1 ± 0.67 B	1.9 ± 0.01 B	441.17± 195.61
P	< 0.0001	< 0.0001	< 0.0001	>0.05
AUG				

	TEMP	HUM	WS	SR
SITE 1<br>SITE 2<br>SITE 3<br>SITE 4	10.2 ± 0.61 A<br>8.9 ± 0.64 B<br>10.5 ± 0.65 A<br>8.7 ± 0.64 B	19.9 ± 0.17 A<br>24.5 ± 0.13 B<br>24.4 ± 0.09 B<br>25.0 ± 0.19 B	2.2 ± 0.06 A<br>2.1 ± 0.06 B<br>1.9 ± 0.06 C<br>2.1 ± 0.06 A	510.55 ± 206.47<br>511.15 ± 206.58<br>511.01 ± 206.53<br>510.82 ± 206.53
P	< 0.0001	< 0.0001	< 0.0001	>0.05
SEP				
	TEMP	HUM	WS	SR
SITE 1<br>SITE 2<br>SITE 3<br>SITE 4	12.5 ± 0.85 A<br>11.3 ± 0.82 B<br>12.9 ± 0.88 A<br>11.1 ± 0.88 B	21.6 ± 0.85 A<br>26.2 ± 0.89 B<br>25.9 ± 0.94 B<br>26.7 ± 0.96 B	2.4 ± 0.02 A<br>2.2 ± 0.02 B<br>2.1 ± 0.02 C<br>2.2 ± 0.02 B	580.21 ± 211.31<br>583.23 ± 208.53<br>581.88 ± 208.29<br>583.08 ± 208.47
P	< 0.0001	< 0.0001	< 0.0001	>0.05
OCT				
	TEMP	HUM	WS	SR
SITE 1<br>SITE 2<br>SITE 3<br>SITE 4	15.3 ± 0.63 A<br>14.2 ± 0.63 B<br>15.7 ± 0.64 A<br>13.9 ±0.63 B	24.8 ± 0.89 A<br>30.0 ± 1.11 B<br>29.8 ± 1.08 B<br>30.5 ± 1.09 B	2.3 ± 0.01 A<br>2.2 ± 0.02 B<br>2.1 ± 0.01 C<br>2.2 ± 0.01 B	618.08 ± 195.17<br>618.33 ± 195.20<br>617.53 ± 195.42<br>618.35 ± 195.12
P	< 0.0001	< 0.0001	< 0.0001	>0.05
NOV				
	TEMP	HUM	WS	SR
SITE 1<br>SITE 2<br>SITE 3<br>SITE 4	16.6 ± 0.16 A<br>15.5 ± 0.15 B<br>17.1 ± 0.15 A<br>15.2 ± 0.15 B	29.2 ± 2.12 A<br>35.3 ± 2.57 B<br>34.9 ± 2.50 B<br>35.9 ± 2.60 B	2.3 ± 0.03 A<br>2.2 ± 0.03 B<br>2.1 ± 0.04 C<br>2.2 ± 0.03 B	620.32 ± 180.02<br>619.24 ± 179.67<br>617.36 ± 179.78<br>619.41 ± 179.60
P	< 0.0001	< 0.0001	< 0.0001	>0.05
DEC				
	TEMP	HUM	WS	SR
SITE 1<br>SITE 2<br>SITE 3<br>SITE 4	16.4 ± 0.16 A<br>15.3 ± 0.18 B<br>16.9 ± 0.18 A<br>15.1 ± 0.18 B	36.9 ± 1.61 A<br>44.7 ± 1.99 B<br>44.1 ± 1.95 B<br>45.4 ± 2.03 B	2.2 ± 0.04 A<br>2.0 ± 0.06 B<br>1.9 ± 0.05 C<br>2.0 ± 0.06 B	563.25 ± 158.11<br>563.65 ± 156.42<br>559.43 ± 155.80<br>563.68 ± 156.65
P	< 0.0001	< 0.0001	< 0.0001	>0.05
