# Supplementary material for: The micro-niche explains allotopy and syntopy in South American Liolaemus (Iguania: Liolaemidae) lizards
Source: PeerJ. 2025 Feb 17;13:e18979. doi: 10.7717/peerj.18979 (PMC11841597; doi:10.7717/peerj.18979)
Supplement: Supplemental Information 2 — A.- Monthly variation of Air Temperature of the four sites studied. Dots refers to mean and lines to standard deviation. B.- Monthly variation of Relative Humidity of the four sites studied. Dots refer to mean and lines to standard deviation. C.- Monthly variation of Wind Speed of the four sites studied. Dots represent the mean, and lines represent the standard deviation. D.- Monthly variation of Sun Radiation of the four sites studied. Dots refers to mean and lines to standard deviation. [file peerj-13-18979-s002.pdf]

Supplementary Figure 1

Monthly variation of the variables studied

A.- Monthly variation of Air Temperature of the four sites studied. Dots refers to mean and lines to standard deviation. B.- Monthly variation of Relative Humidity of the four sites studied. Dots refer to mean and lines to standard deviation. C.- Monthly variation of Wind Speed of the four sites studied. Dots represent the mean, and lines represent the standard deviation. D.- Monthly variation of Sun Radiation of the four sites studied. Dots refers to mean and lines to standard deviation.

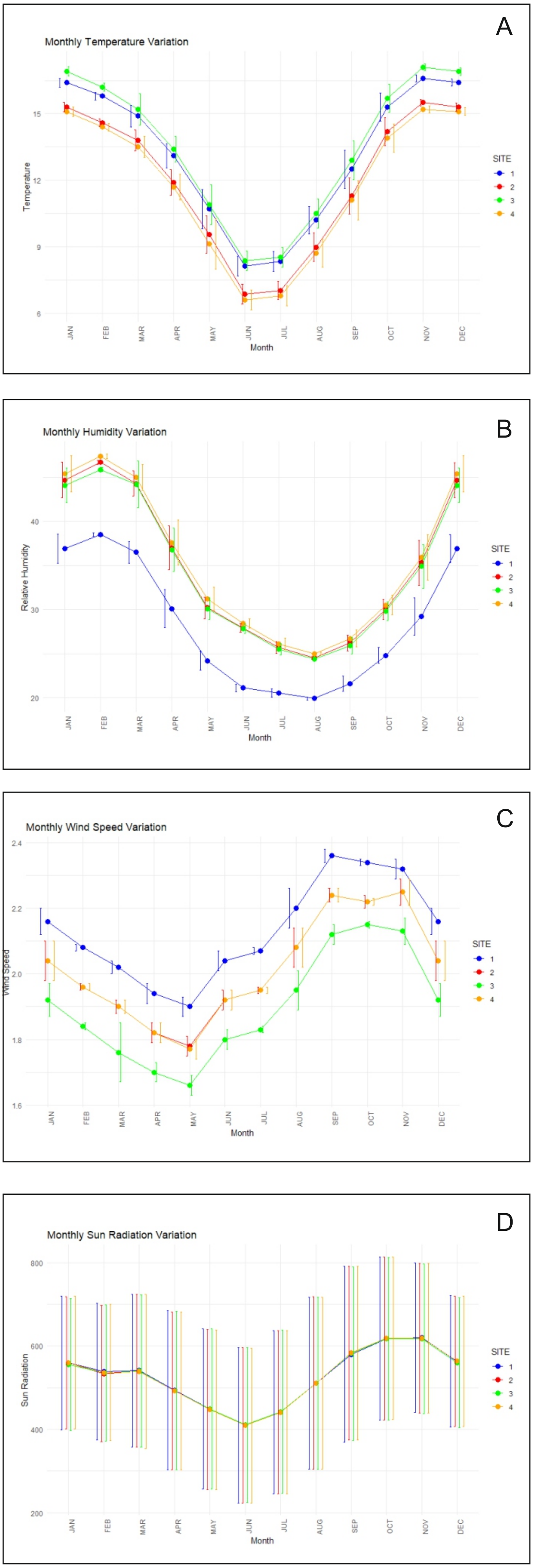
A
Monthly Temperature Variation
Temperature
15
12
9
6
JAN FEB MAR APR MAY JUN JUL AUG SEP OCT NOV DEC
Month
SITE
1
2
3
4
B
Monthly Humidity Variation
Relative Humidity
40
30
20
JAN FEB MAR APR MAY JUN JUL AUG SEP OCT NOV DEC
Month
SITE
1
2
3
4
C
Monthly Wind Speed Variation
Wind Speed
2.4
2.2
2.0
1.8
1.6
JAN FEB MAR APR MAY JUN JUL AUG SEP OCT NOV DEC
Month
SITE
1
2
3
4
D
Monthly Sun Radiation Variation
Sun Radiation
800
600
400
200
JAN FEB MAR APR MAY JUN JUL AUG SEP OCT NOV DEC
Month
SITE
1
2
3
4
