# Supplementary material for: The micro-niche explains allotopy and syntopy in South American Liolaemus (Iguania: Liolaemidae) lizards
Source: PeerJ. 2025 Feb 17;13:e18979. doi: 10.7717/peerj.18979 (PMC11841597; doi:10.7717/peerj.18979)
Supplement: Supplemental Information 3 — Top. - Air Temperature raster, showing hourly variation on month of lizard’s main activity. Color dots identify the sites. Blue: Site 1; Red: Site 2; Green: Site 3; Orange: Site 4. Bottom. - Relative Humidity raster, showing hourly variation on month of lizard’s main activity. Color dots identify sites: Blue: Site 1; Red: Site 2; Green: Site 3; Orange: Site 4. [file peerj-13-18979-s003.pdf]

Supplementary Figure 2

Rasters of Air Temperature and Relative humidity.

Top. - Air Temperature raster, showing hourly variation on month of lizard’s main activity. Color dots identify the sites. Blue: Site 1; Red: Site 2; Green: Site 3; Orange: Site 4. Bottom. - Relative Humidity raster, showing hourly variation on month of lizard’s main activity. Color dots identify sites: Blue: Site 1; Red: Site 2; Green: Site 3; Orange: Site 4.

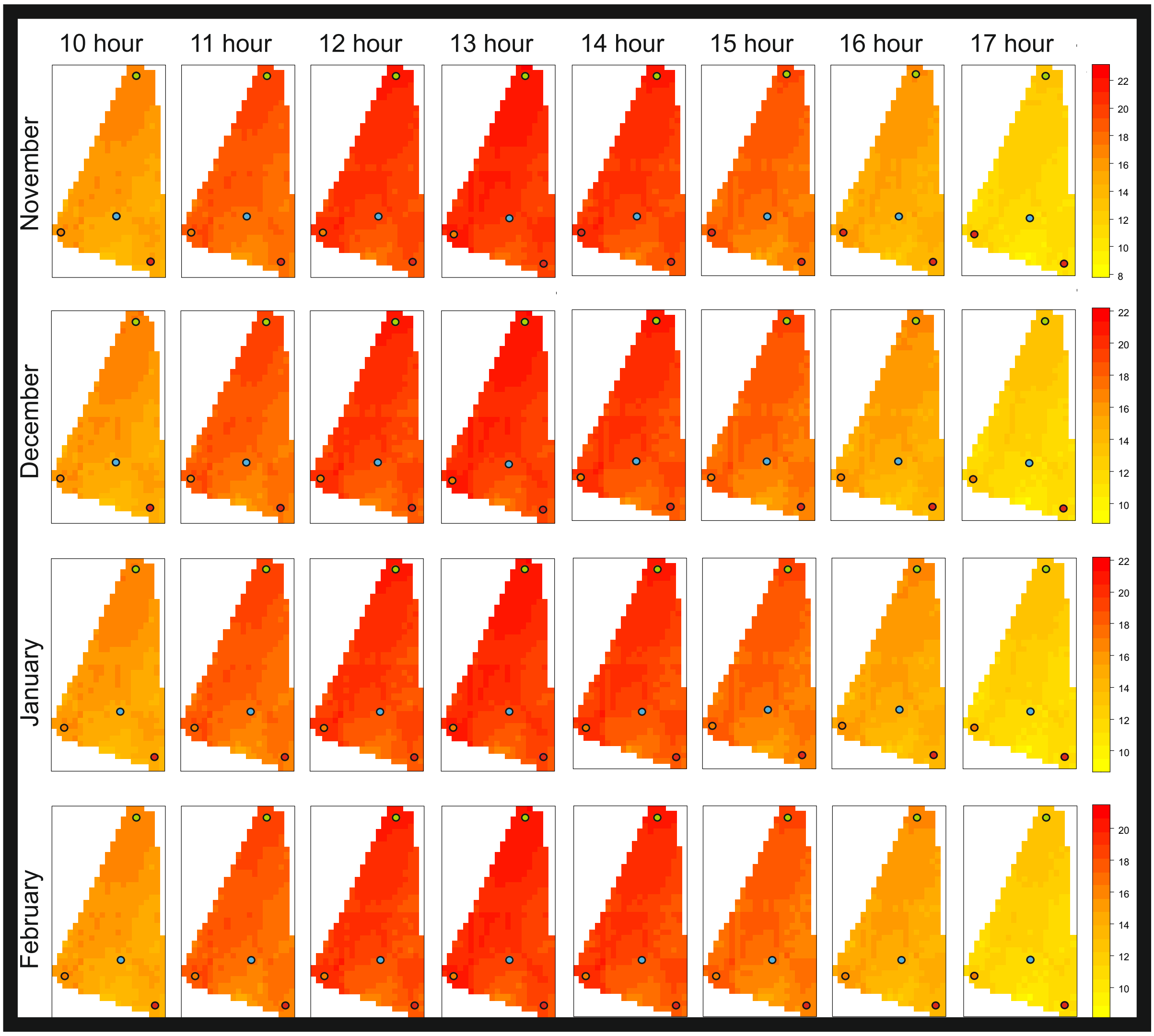
10 hour
11 hour
12 hour
13 hour
14 hour
15 hour
16 hour
17 hour
November
December
January
February
22
20
18
16
14
12
10
8

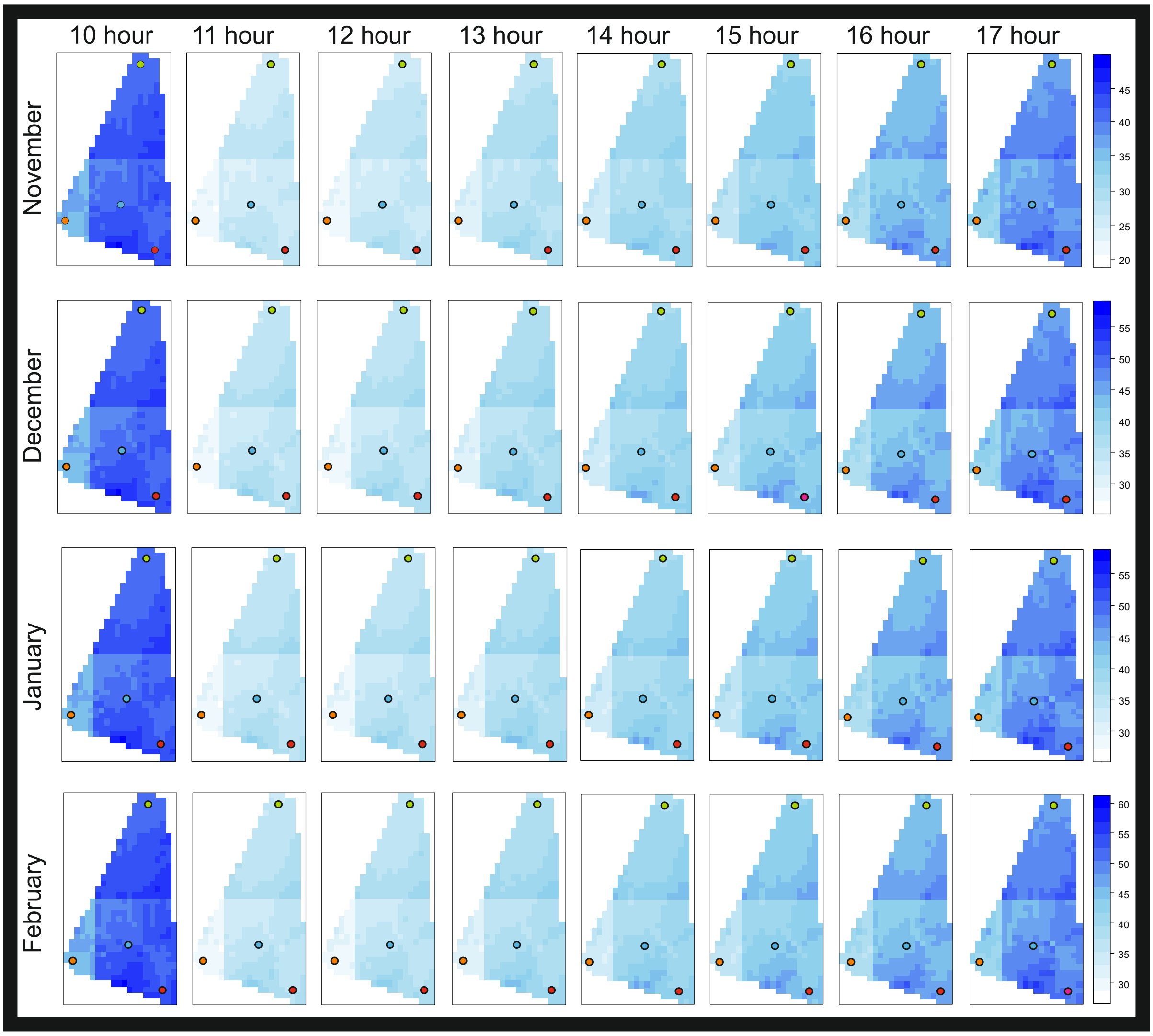
10 hour
11 hour
12 hour
13 hour
14 hour
15 hour
16 hour
17 hour
November
December
January
February
45
40
35
30
25
20
55
50
60
