# Supplementary material for: The micro-niche explains allotopy and syntopy in South American Liolaemus (Iguania: Liolaemidae) lizards
Source: PeerJ. 2025 Feb 17;13:e18979. doi: 10.7717/peerj.18979 (PMC11841597; doi:10.7717/peerj.18979)
Supplement: Supplemental Information 4 [file peerj-13-18979-s004.docx]

Supplementary File 1. Code used for the microclimatic model.

The code was based on Toro Cardona FA, Parra JL, Rojas-Soto, OR. 2023. Predicting daily activity time through ecological niche modeling and microclimatic data. Journal of Animal Ecology, 92, 925–935. https://doi.org/10.1111/1365-2656.13895

install.packages("ncdf4")

library(devtools)

install_github('mrke/NicheMapR')

library(NicheMapR)

library(ncdf4)

vignette("microclimate-model-tutorial", package = "NicheMapR")

help("micro_global")

loc: Longitude and latitude (decimal degrees)

timeinterval: The number of time intervals to generate predictions for over a year (must be 12 <= x <=365)

nyears: The number of years to run

soiltype: Rock = 0, sand = 1, loamy sand = 2, sandy loam = 3, loam = 4, silt loam = 5, sandy clay loam = 6, clay loam = 7, silt clay loam = 8, sandy clay = 9, silty clay = 10, clay = 11, user-defined = 12, based on Campbell and Norman 1990 Table 9.1.

REFL: Soil solar reflectance, decimal %

elev: Elevation, if to be user specified (m)

slope: Slope in degrees

aspect: Aspect in degrees (0 = north)

DEP: Soil depths at which calculations are to be made (cm), must be 10 values starting from 0, and more closely spaced near the surface

minshade: Minimum shade level to use (%)

maxshade: Maximum shade level to use (%)

Usrhyt: Local height (m) at which air temperature, wind speed and humidity are to be computed for organism of interest

nc_data <- nc_open('global_climate.nc')

longlat<-c(-23.30350908, 65.98345656)

mod_1p_1m<-micro_global(loc = longlat, timeinterval =365 , nyears=1, soiltype = 9, Usrhyt = 1 , elev= 2692, slope= 9.39, aspect= 64.9 , minshade = 0, maxshade =48 )

mod_1p_1cm<-micro_global(loc = longlat, timeinterval =365 , nyears=1, soiltype = 9, Usrhyt = 0.01 , elev= 2692, slope= 9.39, aspect= 64.9 , minshade = 0, maxshade =48)

head(mod_1p_1m$metout)

head(mod_1p_1cm$metout)

output_1m<-mod_1p_1m$metout %>% as.data.frame()

output_1cm<-mod_1p_1cm$metout %>% as.data.frame()

write.csv(output_1m, "example.csv")

library(tidyverse)

31+28+31+30+31+30+12

jul12_1m<- output_1m %>% filter(DOY==193)

jul12_1cm<- output_1cm %>% filter(DOY==193)

head(jul12_1cm)

graf<-ggplot()+

geom_line(data=jul12_1cm, aes(x=TIME, y=TALOC), col="black", size=1)+

geom_line(data=jul12_1m, aes(x=TIME, y=TALOC), col="blue", size=1)+

labs(x="Hour", y= "Temperature (°C)")+

theme_bw()

graf

vignette("microclimate-model-theory-equations", package = "NicheMapR")

vignette("microclimate-model-IO", package = "NicheMapR")

vignette("ectotherm-model-tutorial", package = "NicheMapR")

diasmes<-rep(1:12, times =c(31,28,31,30,31,30,31,31,30,31,30,31))

filas <- 12* 24

clima <- data.frame( longitud= rep(NA,filas), latitud=rep(NA,filas) ,Mes=rep(NA,filas), Hora=rep(NA,filas),Tmin=rep(NA,filas),Tmax=rep(NA,filas), Tprom=rep(NA,filas), Hrmin=rep(NA,filas), Hrmax=rep(NA,filas), Hrprom=rep(NA,filas), Wvmin=rep(NA,filas), Wvmax=rep(NA, filas), Wvprom=rep(NA, filas))

ind <- seq(0,dim(clima)[1], 12)

micro_surface <- function(x) {

temp <- micro_global(loc= x[2:3], timeinterval= 365, nyears= 1, soiltype= x[9], elev= x[6], slope= x[7], aspect= x[8], minshade= x[4], maxshade=x[5], Usrhyt= 0.5)$metout

horas <- unique(temp[,2])

meses<-rep(c(1,2,3,4,5,6,7,8,9,10,11,12), times=24, length.out=288)

for(i in 1:24) {

tiempox <- subset(data.frame(temp), TIME==horas[i])

tmean <- tapply(tiempox$TALOC, INDEX=diasmes, FUN=mean)

tmin <- tapply(tiempox$TALOC, INDEX=diasmes, FUN=min)

tmax <- tapply(tiempox$TALOC, INDEX=diasmes, FUN=max)

hrmean <- tapply(tiempox$RHLOC, INDEX=diasmes, FUN=mean)

hrmin <- tapply(tiempox$RHLOC, INDEX=diasmes, FUN=min)

hrmax <- tapply(tiempox$RHLOC, INDEX=diasmes, FUN=max)

wvmean <- tapply(tiempox$VLOC, INDEX=diasmes, FUN=mean)

wvmin <- tapply(tiempox$VLOC, INDEX=diasmes, FUN=min)

wvmax <- tapply(tiempox$VLOC, INDEX=diasmes, FUN=max)

clima[(ind[i]+1):ind[i+1],1] <- x[2]

clima[(ind[i]+1):ind[i+1],2] <- x[3]

clima[(ind[i]+1):ind[i+1],3] <- 1:12

clima[(ind[i]+1):ind[i+1],4] <- horas[i]

clima[(ind[i]+1):ind[i+1],5] <- tmin

clima[(ind[i]+1):ind[i+1],6] <- tmax

clima[(ind[i]+1):ind[i+1],7] <- tmean

clima[(ind[i]+1):ind[i+1],8] <- hrmin

clima[(ind[i]+1):ind[i+1],9] <- hrmax

clima[(ind[i]+1):ind[i+1],10] <- hrmean

clima[(ind[i]+1):ind[i+1],11] <- wvmin

clima[(ind[i]+1):ind[i+1],12] <- wvmax

clima[(ind[i]+1):ind[i+1],13] <- wvmean

}

return(clima)

}

data<-read.csv("D:/ESCUELA AMBIENTAL SIG/Modelos microclimaticos/Data_inpunt_table/Tabla_input.csv", header=T)

head(data)

modelo <- apply(data, 1, micro_surface)

dim(modelo[[1]])

tabla <- do.call('rbind', modelo)

write.table (tabla, file="Modelo_microclim_AF.txt", sep= "\t")

diasmes<-rep(1:12, times =c(31,28,31,30,31,30,31,31,30,31,30,31))

filas <- 12* 24

clima <- data.frame( longitud= rep(NA,filas), latitud=rep(NA,filas) ,Mes=rep(NA,filas), Hora=rep(NA,filas),Tmin=rep(NA,filas),Tmax=rep(NA,filas), Tprom=rep(NA,filas), Hrmin=rep(NA,filas), Hrmax=rep(NA,filas), Hrprom=rep(NA,filas), Wvmin=rep(NA,filas), Wvmax=rep(NA, filas), Wvprom=rep(NA, filas))

ind <- seq(0,dim(clima)[1], 12)

micro_surface <- function(x) {

temp <- micro_global(loc= x[2:3], timeinterval= 365, nyears= 1, soiltype= x[9], elev= x[6], slope= x[7], aspect= x[8], minshade= x[4], maxshade=x[5], Usrhyt= 0.5)$metout

horas <- unique(temp[,2])

meses<-rep(c(1,2,3,4,5,6,7,8,9,10,11,12), times=24, length.out=288)

for(i in 1:24) {

tiempox <- subset(data.frame(temp), TIME==horas[i])

tmean <- tapply(tiempox$TALOC, INDEX=diasmes, FUN=mean)

tmin <- tapply(tiempox$TALOC, INDEX=diasmes, FUN=min)

tmax <- tapply(tiempox$TALOC, INDEX=diasmes, FUN=max)

hrmean <- tapply(tiempox$RHLOC, INDEX=diasmes, FUN=mean)

hrmin <- tapply(tiempox$RHLOC, INDEX=diasmes, FUN=min)

hrmax <- tapply(tiempox$RHLOC, INDEX=diasmes, FUN=max)

wvmean <- tapply(tiempox$VLOC, INDEX=diasmes, FUN=mean)

wvmin <- tapply(tiempox$VLOC, INDEX=diasmes, FUN=min)

wvmax <- tapply(tiempox$VLOC, INDEX=diasmes, FUN=max)

clima[(ind[i]+1):ind[i+1],1] <- x[2]

clima[(ind[i]+1):ind[i+1],2] <- x[3]

clima[(ind[i]+1):ind[i+1],3] <- 1:12

clima[(ind[i]+1):ind[i+1],4] <- horas[i]

clima[(ind[i]+1):ind[i+1],5] <- tmin

clima[(ind[i]+1):ind[i+1],6] <- tmax

clima[(ind[i]+1):ind[i+1],7] <- tmean

clima[(ind[i]+1):ind[i+1],8] <- hrmin

clima[(ind[i]+1):ind[i+1],9] <- hrmax

clima[(ind[i]+1):ind[i+1],10] <- hrmean

clima[(ind[i]+1):ind[i+1],11] <- wvmin

clima[(ind[i]+1):ind[i+1],12] <- wvmax

clima[(ind[i]+1):ind[i+1],13] <- wvmean

}

return(clima)

}

data<-read.csv("D:/ESCUELA AMBIENTAL SIG/Modelos microclimaticos/Data_inpunt_table/Tabla_input.csv", header=T)

head(data)

modelo <- apply(data, 1, micro_surface)

tabla <- do.call('rbind', modelo)

write.table (tabla, file="Modelo_microclim_AF.txt", sep= "\t")

mod1<-read.table("Modelo_microclim_AF.txt", sep= "\t", header = T)

library(dplyr)

unique(mod1$Hora)

jun10<-mod1 %>% filter(Mes==6, Hora==600)

write.csv(example.csv")

library(raster)

ref<-raster("Raster_referencia.tif")

horas<-c(480, 540, 600, 660, 720, 780, 840, 900, 960, 1020, 1080, 1140, 1200)

horas

meses<-unique(mod1$Mes)

meses

ras_k_t<-stack()

tabla_t <- data.frame(mes=rep(c(1:12), each=13), horas=horas,tmin=NA, tmax=NA, tprom=NA)

u=1

for (i in 1:12) {

for (j in 1:length(horas)){

vars_hm_t<-subset(mod1, Mes== meses[i] & Hora== horas[j])

ras_t <- rasterize(vars_hm_t[,1:2], y = ref, field=vars_hm_t$Tprom)

ras_k_t <- addLayer(ras_k_t, ras_t)

u=u+1

}

}

ene480<-subset(mod1, Hora==480 & Mes==1)

max(ene480$Tmax)

min(ene480$Tmax)

plot(ras_k_t[[1]])

summary(ras_k_t[[1]])

dir.create("Tmin_12_8_20h")

writeRaster(ras_k_t, filename ="Tmin_12_8_20h/Tmin_" , bylayer=T, format="GTiff")

library(lattice)

library(latticeExtra)

library(rasterVis)

library(RColorBrewer)

library(raster)

#Example

cols <- colorRampPalette(c(c("#98F5FF", "#FF3030")))

map_mosaic<-levelplot(mic_enero[[c(1:12)]], main="Title", layout=c(3, 4), scales=list(draw=FALSE ), col.regions=cols)

map_mosaic

tiff("Grafico_enero_tmax.tiff", width = 20, height = 25, units = "cm", res=250)

map_mosaic

dev.off()
